# Tubercular Patients on a Hill

**Published:** 1916-04-15

**Authors:** Solali B. Vakil

**Affiliations:** Medical Director. Bel-Air Sanatorium, Panchgani, Bombay Presidency.


*To the Editor of* The Hospital.

### Tubercular Patients on a Hill.

*To the Editor of* The Hospital.

Sir,—Though I have not the honour of personal acquaintance I have ventured to approach you, hoping to be excused and favoured with a reply.

We have started a private sanatorium for consumptives on a small hill, but the municipality objects to its being located there. The opinion of the Royal College of Physicians—"No risk is incurred by living in the immediate neighbourhood of institutions for the treatment of tuberculosis which are properly conducted"—seems not to convince the health officer (who has no diploma in hygiene or public health). The institution has its own compound of thirty acres, the nearest school half a mile away, and house over 200 yards on a steep gradient, so much so that a stranger standing on the verandah of the house would not know that a sanatorium existed there unless he was told so. The hill has only eighty cottages, and it has been the resort of a large number of consumptives staying uncontrolled in private buildings for the last twenty years. No objection was raised then, but now, since a sanatorium has been established, attempts are made to declare it as very dangerous. The management and the supervision have been declared satisfactory by all parties. The only question is the site. As the question is likely to affect several sanatoria in India, we are writing to eminent medical men in India and in England for their views on the subject. May I therefore request you to favour me with your personal opinion in general terms as to the danger of a tuberculosis sanatorium to its vicinity, and whether you will endorse the opinion of the Royal College of Physicians. Would you consider the rate of infectivity of tuberculosis as high as that of smallpox or measles? Your valuable opinion will help a humane cause so much. Thanking you in anticipation.—Yours, etc., Solali B. Vakil,

March 10, 1916. Medical Director.

Bel-Air Sanatorium, Panchgani, Bombay Presidency.

[There is no evidence for the supposition that any danger exists in the vicinity of a tuberculosis hospital. It is quite unreasonable for the inhabitants of the surrounding area to be alarmed at any risk of infection. Even in the wards themselves English experience shows that the risk of infection to nurses and doctors is negligible. Although smallpox may be air-borne in a limited sense, measles probably requires personal contact for its dissemination.—Ed. The Hospital.]

